# Unintended Pregnancies, Their Outcome and Prior Usage of Contraception in Georgia

**Published:** 2017-02

**Authors:** Tamar JAPARIDZE, Jenara KRISTESASHVILI

**Affiliations:** Faculty of Medicine, Ivane Javachishvili Tbilisi State University, Tbilisi, Republic of Georgia

**Keywords:** Unintended pregnancy, Abortion, Contraception, Georgia

## Abstract

**Background:**

Unintended pregnancy (UPr) with high rates of subsequent abortions remains an important problem around the world. Our aim was to determine the prevalence of UPrs in Georgia, their outcomes, and use of modern and traditional methods of contraception in women who terminated their UPrs by induced abortion (IAb) and identify potentially associated factors.

**Methods:**

A Cross-sectional study, using stratified multistage sampling design, was conducted from Oct 2010 through Feb 2011. By secondary data analysis in selected group of women (n=2692) association of predictor variables: area of residence, age, level of education, economic status of woman, ethnicity, and religion with independent variables: UPr, termination of UPr by IAb and prior usage of modern and traditional methods of contraception were examined. Descriptive statistics methods and multivariable logistic regression analysis were used.

**Results:**

In Georgia, from the 2692 interviewed women who had a pregnancy, 1068(39.7%) had a UPr in 2005–2010; the majority (n=913; 85.5%) of these UPr ended in an IAb. 31.4% (n=287) of those women used any method of contraception, with the majority (n=213; 74.2%) using a traditional method. With UPr were associated area of residence, level of education and age (*P*>0.05); with termination of UPr by IAb-ethnic origin (*P*<0.05); with use of contraception –area of residence, age, level of education, ethnicity and religion and with use of modern methods of contraception – age (*P*<0.05).

**Conclusion:**

Low rates of modern contraception use prior UPrs, terminated by IAb demands attention by healthcare providers. Factors associated with these outcomes shall be used to identify target group of women.

## Introduction

UPr with high rates of the abortions remains an important problem around the world. In 2008 out of 210.9 million pregnancies, worldwide 42% were unintended (1). Between 1995 and 2008 the rate of UPrs fell by 20%, to 55 from 69(2). This rate was higher in developed countries with reduction by 29%, to 42 from 59(2), compared to developing countries with reduction by less than 20% - to 57 from 71 per 1000 women(2). High rates of UPrs were in Africa and low rates in Western Europe and Asia. North America was the only region in which overall and UPr rates have not declined (2). The rate of UPrs is determined by specific cultural, traditional, socioeconomic, and health system factors of each country (3, 4). Due to insufficient care for pregnancy UPr may affect sexual, reproductive and general health status of woman, result in serious mental and physical problems for her as well as her family (5).

The problem of UPr is important in Georgia as well. According to the Reproductive Health Survey 2010 in Georgia (6), the rate of UPrs fell by 36% that compared to data from surveys from 1999 and 2005 is by 59% and 51% lower, respectively (7, 8). In dynamics total induced abortions rate (TIAR) have fallen as well. In 1997–1999 it was 3.7 abortion per woman (125 abortions per 1000 women), being one of the highest rates worldwide. The rate of IAbs gradually fell to 3.1 abortions per woman (104 abortions per 1000 women) in 2002–2005, and between 2005 and 2010, the abortion rate dropped significantly to 1.6 abortions per woman (56 abortions per 1000 woman). Given trends can be explained with healthcare system reorganization and with programs conducted by international organizations (United Nations Population Fund (UNFPA), United States Agency for International Development (USAID), United Nations Children’s Fund (UNICEF)) since 2000, leading to improvement of sexual/reproductive health indicators in Georgia (9, 10), although, these indicators are still unfavorable. As a part of the Soviet Union for a long period, Georgia was limited in opportunities to have contacts with Western medicine. In Georgia, in the early 90s, like in other post-Soviet countries, IAb was the main method of birth control (11). IAb was not stigmatized. According to Georgian legislation IAb was, and is at present, legal and is performed based on woman’s will and request until 12 wk of pregnancy (12).

Since 90s of 20^th^ century, the contraception use rate in Georgia was very low and like in other Asian countries, women mainly used traditional methods (withdrawal and rhythm/calendar methods).The contraception prevalence rate for married women increased from 41% in 1999 to 45% in 2005 and 53% in 2010, mainly due to use of modern contraception (6–8). These rates are still insufficient, as the rates of UPrs are still high. Subsequent analysis of 1999 and 2005 Reproductive Health Surveys results showed that non-use of contraception was the major determinant of threat of Abs, and use of modern contraception significantly reduced the number of Uprs (13). The increasing use of modern contraception, especially among young women, significantly reduced the number of UPrs and IAbs.

In order to identify high-risk women and provide implementation of appropriate preventive measures, it is essential for providers of health care system and physicians to know the risk factors associated with UPrs and IAbs.

Objective of our study was to determine the prevalence of UPrs in Georgia, their outcomes, use of modern and traditional methods of contraception in women who terminated their UPr by IAb and identify such predictor variables as area of residence, age, level of education, economic status of woman, ethnicity and religion.

## Materials and Methods

Secondary analysis of database of 2010 Reproductive Health Survey was performed. This cross-sectional study, using stratified multistage sampling method was conducted from Oct 2010 through Feb 2011, with approval of Ethics Committee of National Center for Disease Control of Georgia. After obtaining informed consent 6292 women regardless of marital status, who lived in Georgia during the survey period (excluding separatist regions of Abkhazia and South Ossetia) were interviewed at their homes. The inclusion criterion was age of 15 and 44. In addition, for our study, inclusion criteria were: for identifying predictor variables for UPr - women, who had a pregnancy within last 5 yr; for termination of unplanned pregnancy by IAb- women having unplanned pregnancy, for use of contraception - women, who terminated UPr by IAb and for use of modern and traditional methods of contraception - use of any method of contraception. Anyone outside these groups was excluded from the analysis.

The survey helped to obtain data about basic characteristics and health indicators of population. Based on the responses received through the survey four independent variables have been selected: UPr, termination of UPr by IAb, use of contraception and use of modern or traditional methods of contraception in women who terminated their UPr by IAb.

Women, who were unwilling to become pregnant or planned to become pregnant later were included in the category of UPr; and women who were willing to become pregnant or were uncertain in their intentions were included in the category of intended pregnancy.

Information about the outcome of a pregnancy – termination by IAb or prolongation of pregnancy – was obtained from responses to the question how their UPrs ended. Women with “induced abortion” (sharp curettage above 5wk of pregnancy) and “mini-abortion” (vacuum aspiration under 5 wk of pregnancy) were regarded as a category of IAb, and others were classified as women having a pregnancy (delivery and several cases of ectopic pregnancy).

Women were also asked whether they used any method of contraception before they got pregnant with this pregnancy and what method of contraception was used. Modern methods of contraception included use of the pill, intrauterine device (IUD), condom plus IUD, condom plus traditional method of contraception, spermicides, or injectable, while traditional methods of contraception included use of calendar/rhythm method, withdrawal, and calendar plus withdrawal. Data analysis was performed in relation to either use of modern methods of contraception or traditional methods of contraception.

In our study, the factors potentially associated with UPr, its outcome and use of contraception included: area of residence, age, level of education, material status of woman, ethnic origin, and religion.

Data were analyzed using the statistical software SPSS ver. 17. Multinomial logistic regression was done to calculate odds ratios (ORs) for intended and unintended pregnancies, their outcomes and use of contraception, including modern or traditional methods for area of residence, age, level of education, ethnicity, material status and religion.

## Results

### Descriptive statistical analysis

Average age of women involved in the survey was 29.5 (SD=8.1; range=15–44). Overall, 2692 respondents (42.8%) reported having pregnancies within last 5 years. Majority were ethnically Georgian Orthodox women. 38% had university/postgraduate education, more than half lived in rural areas and more than average number of respondents believed that had more or less satisfactory economic condition (Table 1). Of 1068(39.7%), women reported that their pregnancies were unintended: 303(28.4%) had a mistimed pregnancy and 765(71.6%) had an unwanted pregnancy. Overall, 913(85.5%) of them ended the pregnancy by IAb. Totally, 626(68.6%) did not use any method of contraception, 287(31.4%) used any method of contraception, and 74(25.8%) of them used modern methods of contraception.

**Table 1:** Background characteristics of study sample (n=2,692)

**Variable**	**n**	**(%)**
**Pregnancy intention**		
Intended	1624	60.3
Unintended	1068	39.7
**Residence**		
Urban	1221	45.4
Rural	1471	54.6
**Age (yr)**		
15–19	69	2.6
20–24	530	19.7
25–29	757	28.1
30–34	681	25.3
35–39	439	16.3
40–44	216	8.0
**Education**		
Incomplete secondary	447	16.6
Secondary	696	25.9
Technical	523	19.4
Diploma/Postgraduate	1026	38.1
**Economic status of the family**		
Can hardly satisfy their needs	788	29.3
Can somehow satisfy their needs	1758	65.3
Can easily satisfy their needs	146	5.4
**Ethnicity**		
Georgian	2292	85.1
Other	400	14.9
**Religion**		
Georgian Orthodox	2217	82.4
Other orthodox	145	5.4
Muslim	268	10.0
Other religion	42	1.6
No religion	20	0.7

### Multinomial logistic regression analysis Unintended pregnancy and induced abortion

Multinominal logistic regression analysis showed that higher risk of UPrs had women living in rural areas, compared to those living in urban locations. At the same time, the lower level of education, lower economic condition and higher age of woman, higher is the risk of UPr. After adjusting for these variables, level of education and age remained associated with the risk of UPrs (Table 2). Ethnic origin (*P*=0.173) and religion (*P*=0.191) have no impact on the rates of UPrs. Regarding termination of pregnancy by IAb, no variable was found to have statistically significant association (*P*>0.05) except ethnic origin. In case of UPrs Georgian women were less likely to terminate the pregnancy by IAb (OR=0.563; 95% CI = 0.326–0.971; *P*=0.039) compared to women of another ethnic origin.

**Table 2:** Odds ratios (ORs) of having unintended pregnancy by demographic characteristics

**Variable**	**OR Crude**	**95%CI**	**OR adjusted**	**95%CI**
**Residence**				
Urban	1.382***	1.182–1.616	1.131	0.947–1.350
Rural	1		1	
**Age (yr)**				
15–19	7.621***	3.923–14.808	11.932***	6.049–23.537
20–24	5.500***	3.910–7.736	6.595***	4.626–9.401
25–29	3.765***	2.743–5.169	4.109***	2.967–5.691
30–34	2.130***	1.553–2.921	2.166***	1.567–2.994
35–39	1.508*	1.079–2.108	1.515*	1.076–2.134
40–44	1		1	
**Education**				
Diploma/Post graduate	2.044***	1.629–2.565	2.373***	1.824–3.087
Technicum	1.423**	1.103–1.836	1.598**	1.211–2.108
Secondary	1.276*	1.005–1.619	1.161	0.901–1.497
Incomplete secondary	1		1	
**Economic status of the family**				
Can hardly satisfy their needs	2.404***	1.624–3.559	1.583*	1.040–2.408
Can somehow satisfy their needs	1.454***	1.227–1.724	1.103	0.914–1.330
Can easily satisfy their needs	1		1	

The reference category is unintended pregnancy.

*** (*P*<0.001),

** (*P*<0.01),

* (*P*<0.05).

### Use of contraception

With regards to use of any method of contraception no statistically significant difference was found according to area of residence (*P*=0.825), age (*P*=0.494), level of education (*P*=0.981), material status (*P*=0.795) and ethnicity (*P*=0.237). Non-Georgian Orthodox women were more likely to use any method of contraception compared to Orthodox Georgian women (OR=2.015; 95% CI = 1,175–3.457; *P*=0,011).

Data analysis of women who ended their UPrs by IAb and used any method of contraception (n=287) showed that majority used traditional methods of contraception (n=213, 74.2%). Logistic regression analysis of use of traditional methods of contraception showed that type of contraception statistically significantly differed according to area of residence, age, level of education, ethnic origin and religion (Table 3).After adjusting for these variables, only age was associated with the usage of modern methods of contraception. Women of 20–24yr (OR=11.152; 95% CI=3.030–41.044; *P*=0.001), 25–29yr (OR=3.594; 95% CI=1.052–12,277; *P*=0.041), 30–34yr (OR=4.870; 95%CI= 1.484–15,981; *P*=0.009), and 35–39yr(OR=3.713; 95% CI = 1.107–12,454; *P*=0.034) age groups were more likely to use modern methods of contraception compared to women of 40–44 age group.

**Table 3:** Odds ratios (ORs) of usage of methods of contraception by demographic characteristics

**Variable**	**OR Crude**	**95%CI**
**Residence**		
Urban	2.716***	1.580–4.671
Rural	1	
**Age (yr)**		
15–19	5.000	0.367–68.080
20–24	8.824**	2.552–30.501
25–29	3.636*	1.121–11.791
30–34	4.000*	1.279–12.514
35–39	2.909	0.904–9.363
40–44	1	
**Education**		
Diploma/Post graduate	4.000**	1.746–9.164
Technicum	2.067	0.830–5.150
Secondary	1.032	0.409–2.600
Incomplete secondary	1	
**Ethnicity**		
Georgian	4.233*	1.619–11.072
Other	1	
**Religion**		
Georgian Orthodox	1.00	
Other orthodox	0.285*	0.083–0.981
Muslim	0.152*	0.035–0.655
Other religion	-	-
No religion	-	-

The reference category is traditional.

*** (*P*<0.001),

** (*P*<0.01),

* (*P*<0.05)

## Discussion

The impacts of various factors women have different ORs concerning UPrs, termination of UPrs and use of contraception in pre-abortion periods.

The risk of UPr is lower in women with higher level of education, as due to career advancement women prefer to become pregnant in later ages (14–17). Thus, intension to avoid unwilling pregnancy motivates her to search for and use safe and highly effective methods of contraception. They have high probability of acquiring knowledge about sexual health, providing them with more autonomy and ability to make a decision.

Based on the traditions in the country attitude of women towards pregnancy differs by age. In some countries, risk of UPrs is associated with lower age (18). The risk of UPr increases with age. Young women begin sexual life mainly after marriage and want to become pregnant and have a child very soon. Therefore, most of pregnancies in young age are intended and accordingly there is a low demand for contraception (19). Besides, young women, who have higher reproductive potential, are more motivated to use modern methods of contraception, especially condoms. Along with potential increase of the rate of UPrs in women of higher age, frequency of the use of modern methods of contraception is low, as women tend to practice previous experience of using traditional, less effective contraception, which also increases the risk of UPrs.

Based on socio-economic conditions and differences of tradition of various countries area of residence of woman has different impact on the rate of UPrs (20). In Georgia, women living in rural areas have lower accessibility to education and health services, limiting them to obtain complete information/knowledge about modern effective methods of contraception and use them; therefore, they have higher risk of UPrs. Women with poor economic condition have a problem with obtaining of sufficient knowledge about use of contraception. This leads to contraceptive failure, resulting in increase of the risk of UPrs, with no differences of use of contraception.

The vast majority (85.5%) of UPrs ended by IAb that significantly exceeds the indicators in different regions of the world: 53% in developed countries and 48% in developing countries (2). This finding might reflect a combination of the widespread desire for small families (TFR in Georgia 2.0 per women), low prevalence of modern contraception and the abortion still remains as the main method of birth control (6). Therefore, decrease in the rate of abortions should be mainly achieved through reduction of the number of UPrs.

Factors associated with UPrs have been identified, although none of them, except ethnicity, was associated with IAbs. Presumably, the decision to terminate pregnancy by IAb is influenced by other factors such as previous IAbs, number of livebirths, marital status, attitude of partner/husband, etc. In case of UPr women with higher level of education prefer to terminate it by abortion (21). At present, the rate of abortions did not differ in women with different levels of education.

Effective modern contraception is an alternative way to control UPrs by IAb. In Georgia, like in other Asian countries (22), in pre-abortion period women with UPr mainly used less effective traditional methods of contraception (74.2%) compared to use of modern methods (68.6%). Despite, there were no barriers in Georgia in terms of purchasing the methods of contraception (they could get at the pharmacies modern methods of contraception without doctor’s prescription). This indicates insufficient knowledge about modern methods of contraception. Apparently, health care professionals, mass media, and social contacts provide better informing of women about available choices in contraception, thus promoting increased use of modern methods of contraception, which is in full compliance with recommendations and experiences of studies carried out in other countries (23, 24). Based on experience gained in Netherlands, increasing awareness on contraception in younger ages is especially important (25).

## Conclusion

This study identified factors associated with UPrs, their termination and use of contraception in Georgia. That indicates on the necessity of development of such strategy implementation, which provides improvement contraception knowledge, especially modern methods, increase accessibility to and use of these methods. This, in turn, will reduce the number of UPrs and associated IAbs with direct participation of health care providers. We suggest consideration of these factors in Georgia and intensify work with those target groups, who are at high risk of UPr, particularly, women living in rural areas, women of older age, those with low level of education and poor economic condition.

## Ethical considerations

Ethical issues (Including plagiarism, informed consent, misconduct, data fabrication and/or falsification, double publication and/or submission, redundancy, etc.) have been completely observed by the authors.
